# Enhanced Gut-Homing Dynamics and Pronounced Exhaustion of Mucosal and Blood CD4^+^ T Cells in HIV-Infected Immunological Non-Responders

**DOI:** 10.3389/fimmu.2021.744155

**Published:** 2021-10-07

**Authors:** Kristina Berg Lorvik, Malin Holm Meyer-Myklestad, Kushi Kushekar, Charlotte Handeland, Asle Wilhelm Medhus, Marius Lund-Iversen, Birgitte Stiksrud, Dag Kvale, Anne Margarita Dyrhol-Riise, Kjetil Taskén, Dag Henrik Reikvam

**Affiliations:** ^1^ Department of Infectious Diseases, Oslo University Hospital, Oslo, Norway; ^2^ Department of Cancer Immunology, Institute for Cancer Research, Oslo University Hospital, Oslo, Norway; ^3^ Centre for Molecular Medicine Norway, Nordic EMBL Partnership, University of Oslo, Oslo, Norway; ^4^ Institute of Clinical Medicine, University of Oslo, Oslo, Norway; ^5^ Department of Gastroenterology, Oslo University Hospital, Oslo, Norway; ^6^ Department of Pathology, Oslo University Hospital, Oslo, Norway

**Keywords:** HIV, immunological non-responder, PD1, CD4 T cell, TIGIT, gut-homing CD4^+^ T cell, lamina propria, exhaustion

## Abstract

Immunological non-responders (INR), a subgroup of people living with HIV (PLHIV) who fail to restore CD4^+^ T cell numbers upon effective antiretroviral treatment, have impaired gut mucosal barrier function and an inferior clinical prognosis compared with immunological responders (IR). The contribution of gut-homing and exhaustion of mucosal T cells to the INR phenotype was previously unknown. Flow cytometry analysis of mononuclear cells from peripheral blood and ileal and colonic lamina propria showed that INR had higher fractions of gut-homing CD4^+^ T cells in blood compared with IR. In addition, gut-homing cells were more likely to display signs of exhaustion in INR. The increased CD4^+^ T cell exhaustion in INR was ubiquitous and not restricted to subpopulations defined by activation, differentiation or regulatory T cell markers. In INR, colon CD4^+^ T cell exhaustion correlated negatively with the fraction of CD4^+^ T cells in the same compartment, this was not apparent in the ileum. The fraction of exhausted mucosal CD4^+^ T cells correlated with I-FABP and REG3α, markers of enterocyte damage. We conclude that alterations of gut-homing and exhaustion of T cells may contribute to impaired gut immune and barrier functions associated with immunological non-response in PLHIV.

## Introduction

The gastrointestinal tract is the major site of HIV infection and replication. Early after infection there is a massive depletion of mucosal CD4^+^ T cells and a disruption of the epithelial layer that serves as a protective barrier against luminal content (1, 2). The consequence is typically translocation of microbial products from the gut lumen into the lamina propria and gut-associated lymphoid tissue (GALT), with a subsequent systemic inflammation characterized by an increase in inflammatory mediators and activation of immune cells (3–5). Upon effective antiretroviral therapy (ART), circulating CD4^+^ T cells are restored and the level of systemic inflammation is reduced, but not normalized. The restoration of gut CD4^+^ T cells is slower than in the peripheral blood and it is under debate how effective ART is in restoring mucosal immunity (6). Selective depletion of CD4^+^ T cells and expansion of CD8^+^ T cells in HIV infection leads to a low CD4:CD8 ratio, which is associated with increased risk of non-AIDS morbidity and mortality in virally suppressed people living with HIV (PLHIV) (7). Altered recruitment, exhaustion, and differentiation of CD4^+^ T cells are factors that could hamper normalization of the mucosal immunity in PLHIV (8–11).

In 12-30% of ART-treated PLHIV, the CD4^+^ T cells do not sufficiently recover despite full viral suppression (12, 13). This group of PLHIV, commonly termed immunological non-responders (INR), has increased chronic inflammation, immune activation, and risk of non-AIDS related adverse events such as cardiovascular disease, malignancies, and death (7, 14–17). The etiology of the INR phenotype remains enigmatic but a prevailing hypothesis is that a dysfunctional mucosal barrier impairs CD4^+^ T-cell homeostasis (4, 18).

The surface receptor integrin α4β7 serves as a gut-homing marker as it enables T cells to migrate into mucosal effector sites by binding to mucosal addressin cell adhesion molecule-1 (MAdCAM-1) expressed on mucosal endothelial cells (19). Reports on α4β7 expression and T cell gut-homing dynamics in INR are scarce. Higher frequencies of circulating α4β7^+^ CD4^+^ T cells in INR have been reported (20, 21) but it has not been investigated whether this correlates with enterocyte damage or alterations in gut immunity of INR.

T cell exhaustion is proposed to contribute to the incomplete recovery of CD4^+^ T cells in PLHIV (22). Expression of exhaustion markers, such as programmed death-1 (PD1) and T cell immunoreceptor with Ig and ITIM domains (TIGIT), indicate reduced functional and proliferative capacity of T cells (23, 24). In blood, INR have more exhausted T cells (25, 26) and exhibit a more differentiated and activated T cell phenotype than IR (17, 27, 28). Whether exhaustion in INR is restricted to certain differentiation stages or activation status of T cells remain unknown. Furthermore, T cell exhaustion in gut mucosal tissue of PLHIV is yet to be assessed. Resolving these issues is of great importance in deciphering the disease mechanisms responsible for the insufficient immune recovery in INR and can identify new therapeutic targets for adjuvant treatment and improved clinical prognosis.

Well-defined study groups and extensive mucosal sampling are rare in human INR studies. In this study, we present data from *two* gut anatomical sites in addition to blood. We hypothesized that INR have altered mucosal recruitment of CD4^+^ T cells and that INR have higher levels of exhausted mucosal T cells, which would be restricted to differentiation and activation and associated with enterocyte damage. Testing these hypotheses adds valuable information on how T cell responses can be modulated in order to restore the CD4^+^ T cell numbers and enhance mucosal immunity in INR.

## Materials and Methods

### Participants and Study Design

In total, 39 Caucasian men aged 25-65 were recruited at the out-patient clinic of Department of Infectious Diseases, Oslo University Hospital, Oslo, Norway. The participants and study design have been presented in a previously published paper (29). Briefly, inclusion criteria were HIV seropositive and on continuous ART for > 4 years, and < 50 copies/ml of HIV RNA in blood for > 3.5 years. Based on CD4 count, these subjects were included into two groups: INR (CD4 count < 400 cells/µl for > 3.5 years, N = 19), and IR (CD4 count > 600 cells/µl for > 3.5 years, N = 20). HIV seronegative Caucasian men, referred to the Department of Gastroenterology for control after polyp removal, were recruited as HIV negative controls (HIV-, N = 20). All three groups were matched on age and PLHIV were in addition matched on nadir CD4 count. Inclusion and exclusion criteria can be found in the report by Meyer-Myklestad et al. (29).

Written informed consent was obtained from all participants. The study was approved by the Regional Committee for Medical and Health Research Ethics (approval id 2015/2125) and conducted according to the Declaration of Helsinki.

### Sample Collection

Phlebotomy was performed, and the blood was collected into BD Vacutainer^®^ CPT™ tubes for direct separation of peripheral blood mononuclear cells (PBMC). After centrifugation at 1750 rcf for 17 min at ambient room temperature, PBMC were isolated, put in heat-inactivated fetal calf serum (FCS) with 25% Roswell Park Memorial Institute 1640 (RPMI) and 10% DMSO, and cryopreserved at -150°C until analysis.

Biopsies from the lamina propria of the terminal ileum and the sigmoid colon were taken during colonoscopy. 20 pinch biopsies from the terminal ileum and 20 pinch biopsies from the sigmoid colon were pooled into two separate 50-ml Falcon tubes containing 30 ml ice-cold RPMI with L-glutamin supplemented with 10% FCS, 1% Penicillin/Streptomycin and, respectively (complete medium). After 45 min, the tissue was put in 10 ml pre-heated RPMI w/10% FCS, 10 mg collagenase type H (Cat.# C8051, Sigma-Aldrich, Darmstadt, Germany) and 1.5 µl DNase I (Cat.# 18047019, Invitrogen, Carlsbad, CA) and put on a shaker at 250 rpm and 37°C for 40 min. Next, the tissue was dissolved by mixing with a 18G blunt needle on a 20 ml syringe. All subsequent work was performed on ice/at 4°C. The mucosal cell suspension was filtered through a 70 µm strainer and washed twice. Cell number and viability was determined before 3-4 x10^6^ cells were aliquoted into cryotubes and frozen in FCS w/25% RPMI and 10% DMSO at -150°C until analysis.

### Flow Cytometry Analysis of Cell Surface Markers

PBMC were thawed, washed and resolved in 2 ml complete medium. The cells were transferred into one well in a 24-well plate and rested overnight in a 5% CO2-enriched, humidified incubator at 37°C. Next, the cells were washed twice in phosphate-buffered saline (PBS) with 2% FCS. Cell concentration and viability were determined, before 5x10^5^ cells were stained for 30 min at room temperature protected from light with the following monoclonal antibodies: anti-CD3-PerCP (clone UCHT1, BioLegend), anti-CD4-AF700 (clone SK3, BioLegend), anti-CD38-AF488 (clone HIT2,BioLegend), anti-HLA-DR-BV711 (clone G46-6, BD Biosciences), anti-CD45RA-APC-H7 (clone HI100, BD Biosciences), anti-CD27-BV510 (clone L128, BD Biosciences), anti-PD1-BV785 (clone EH12.2H7, BioLegend), anti-TIGIT-BV421 (clone A15153G, BioLegend), anti-integrin (ITG) β7-APC (clone FIB504, BioLegend), and 7AAD (BD Biosciences).

Mucosal cells were thawed and treated with complete medium with 30 U/ml DNase I for 1 minute, filtered through 70 µm filters and washed twice. The cells were resuspended in 2 ml complete medium and put in tilted 15-ml tubes with a loose cap to rest overnight in a 5% CO2-enriched humidified incubator at 37°C. The next day, the samples were washed in PBS ^+^ 2% FCS, treated with 1.25 µg human BD Fc block (BD Biosciences, cat.# 564220) in PBS with 2% FCS for 10 min in room temperature, before being stained with the following monoclonal antibodies: anti-CD3-PerCP (clone UCHT1, BioLegend), anti-CD8a-AF700 (clone RPA-T8, BioLegend), anti-CD38-AF488 (clone HIT2,BioLegend), anti-HLA-DR-BV711 (clone G46-6, BD Biosciences), anti-CD45RA-APC-H7 (clone HI100, BD Biosciences), anti-CD27-BV510 (clone L128, BD Biosciences), anti-PD1-BV785 (clone EH12.2H7, BioLegend), anti-TIGIT-BV421 (clone A15153G, BioLegend), anti-ITGβ7-APC (clone FIB504, BioLegend), anti-EpCAM-BV650 (clone 9C4, BioLegend), anti-CD127-PE-Cy7 (clone eBioRDR5, eBioscience/Thermo Fisher), anti-CD25-PE (clone 4E3, MACS Miltenyi Biotec) and 7AAD (BD Biosciences). The samples were acquired on a BD LSR Fortessa flow cytometer equipped with four lasers. The flow cytometry data was analyzed with the FlowJo software (version 10, BD). Gating strategy and use of technical controls can be found in **Figure S1** for PBMC and **Figure S2** for mucosal cells.

### Soluble Markers

Serum and plasma tubes were centrifuged according to manufactures’ instruction, frozen at -80°C, thawed in 37°C for 5 minutes before analysis at room temperature. Commercial enzyme-linked immunosorbent assays were used for duplicate analysis of intestinal fatty acid binding protein (I-FABP) in serum (Hycult Biotech, Uden, The Netherlands; CV 8.2%) and human regenerating islet-derived protein 3α (REG3α) in EDTA plasma (R&D Systems, Abington, Oxon, UK; CV 3,2).

### Immunohistochemistry

Single pinch biopsies from the terminal ileum and sigmoid colon were fixed in formaldehyde for 24 hours, transferred to PBS and stored at 4°C until automatic processing of the tissue for molding in paraffin blocks. Immunohistochemistry was performed on a random selection of nine study subjects (three from each group) to analyze the expression of PD-ligand 1 (PD-L1) in both terminal ileum and sigmoid colon (a total of 18 samples). 3 µm thin sections were treated in PT-link with FLEX Target Retrieval Solution, High pH (pH 9.0) (Cat.# K8004, DAKO, Agilent). EnVision FLEX Peroxidase-Blocking Reagent (0.03% H2O2) was used for inhibition of endogenous peroxidase (Cat.# K8024, DAKO, Agilent). The slides were next stained with anti-PD-L1 (405.9A11) mouse monoclonal antibody (Cat.# 29122, Cell Signaling Technology, Danvers, MA, USA, clone 1543a). Secondary antibody conjugation EnVision Flex DAB^+^ mouse (Cat.# K8010, K8012, Dako, Agilent) was used as detection system. Tissue from tonsil was used as a positive staining control (**Figure S3**). PD-L1 expression was determined by visual assessment. Slides with less than one percent positive cells were defined as negative. For tissue orientation, a second set of tissue sections from each sample was stained with hematoxylin and eosin. Microscopic evaluation was performed with Nikon eclipse 80i with Nikon Plan Fluor lenses. Images were captured with Nikon digital sight DS-U3 using Nikon imaging software (NIS) elements, version 4.60. Final adjustments of white balance correction and basic level adjustments were done in Photoshop CC 2018. The proportion of PD-L1 positive cells was manually and blindly determined by an experienced pathologist (M.L.I).

### Statistical Analyses

Data were assumed to not be normally distributed and non-parametric statistics were applied. The three experimental groups were compared by Kruskal-Wallis test with Dunn’s post-test between INR and IR. Statistical analyses were performed using Prism 8 (GraphPad Software, La Jolla, CA).

## Results

### Study Participants’ Characteristics

Blood samples and mucosal biopsies from sigmoid colon and terminal ileum of 19 INR and 20 IR PLHIV who were all virally suppressed by ART, as well as 20 HIV negative healthy controls, were analyzed. The cohort has recently been presented (29). Briefly, all participants were Caucasian men matched by age, and PLHIV were in addition matched on nadir CD4 count. At inclusion, INR and IR had median blood CD4 counts of 327 cells/µL and 777 cells/µL respectively and INR had a lower median of blood CD4:CD8 T cell ratio than IR (0.48 *vs*. 1.00). There were no significant differences between the PLHIV groups with regards to duration of continuous ART, CD8^+^ T cell count at inclusion, and body mass index (29).

### INR Have Higher Fractions of Gut-Homing CD4^+^ T Cells in Blood Than IR

**Figure 1:**
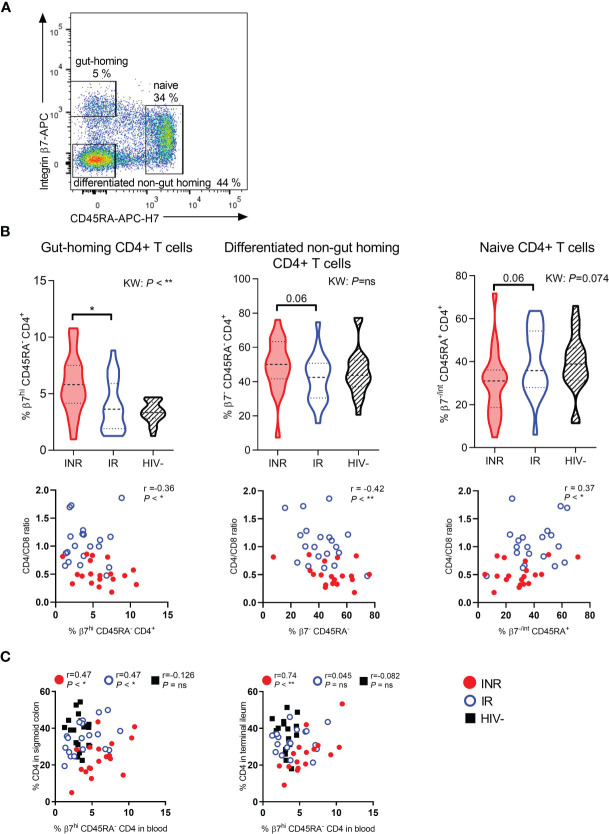
INR have higher fractions of gut-homing CD4^+^ T cells in blood correlating with mucosal CD4^+^ T cell fractions. **(A)** Representative flow cytometry plot of gut-homing (integrin β7^high^ CD45RA^neg^), differentiated non-gut homing (integrin β7^neg^ CD45RA^neg^), and naive (integrin β7^neg/int^ CD45RA^pos^) CD4^+^ T cells in blood. **(B)** Upper part: Comparison of the percentages of gut-homing (left panel), differentiated non-gut homing (middle panel), and naive (right panel) CD4^+^ T cells in immunological non-responders (INR), immunological responders (IR) and HIV negative donors. Lower part: Correlation between CD4:CD8 ratio and gut-homing (left), differentiated non-gut homing (middle), and naive (right) CD4^+^ T cells. **(C)** Correlation between the percentage of CD4^+^ T cells in sigmoid colon (left) and terminal ileum (right) and gut-homing CD4^+^ T cells in blood. INR indicated as red violin plots/dots, IR as open blue violin plots/circles, HIV negatives as lined black violin plots/black squares (dashed lines: median, dotted lines: quartiles). Statistical analyses were performed by Kruskal Wallis (KW) test with Dunn’s post-test between INR and IR and Spearman correlation. Spearman computed on all data combined or on each distinct study group. *P < 0.05, **P < 0.01, ns, not significant.

To investigate whether INR have more gut-homing CD4^+^ T cells, we analyzed ITGβ7 stained PBMC by flow cytometry and included anti-CD45RA to make a crude separation between naive (CD45RA^+^) and differentiated (CD45RA-) CD4^+^ T cells (**Figure 1A**). We found higher percentages of gut-homing (ITGβ7^hi^ CD45RA^-^) CD4^+^ T cells in the blood of INR compared to IR (*P* < 0.05, **Figure 1B**, upper left panel). There was also a tendency of higher fractions of differentiated non-gut homing CD4^+^ T cells (ITGβ7^-^ CD45RA^-^, differentiated cells with a homing potential elsewhere than the gut) and lower fractions of naive (ITGβ7^-/int^ CD45RA^^+^^) CD4^+^ T cells in INR than in IR (**Figure 1B**, upper middle and right panels). The fractions of both gut-homing and non-gut homing differentiated CD4^+^ T cells correlated negatively with blood CD4:CD8 ratio (r=-0.36, *P* < 0.05 and r=-0.42, *P* < 0.01, respectively, **Figure 1B**, lower left and middle panels). The fraction of naive CD4^+^ T cells correlated positively with the CD4:CD8 ratio (r=0.37, *P* < 0.05, **Figure 1B**, lower right panel).

We further studied mucosal cells and investigated whether circulating gut-homing CD4^+^ T cells were linked to the prevalence of CD4^+^ T cells in the gut lamina propria. In the sigmoid colon, a positive correlation between these two T cell subsets was found for both INR and IR (r=0.47, *P* < 0.05, and r=0.47, *P* < 0.05, respectively), but not for the HIV negative control group (**Figure 1C**). Interestingly, for INR there was also a strong positive correlation between the fractions of gut-homing CD4^+^ T cells in blood and the fractions of lamina propria CD4^+^ T cells in terminal ileum (*P* < 0.01, r=0.74), which was not evident for the two other groups (**Figure 1C**).

### INR Have More Exhausted CD4^+^ T Cells in Blood and Gut, Which Both Correlate With Markers of Enterocyte Damage

**Figure 2:**
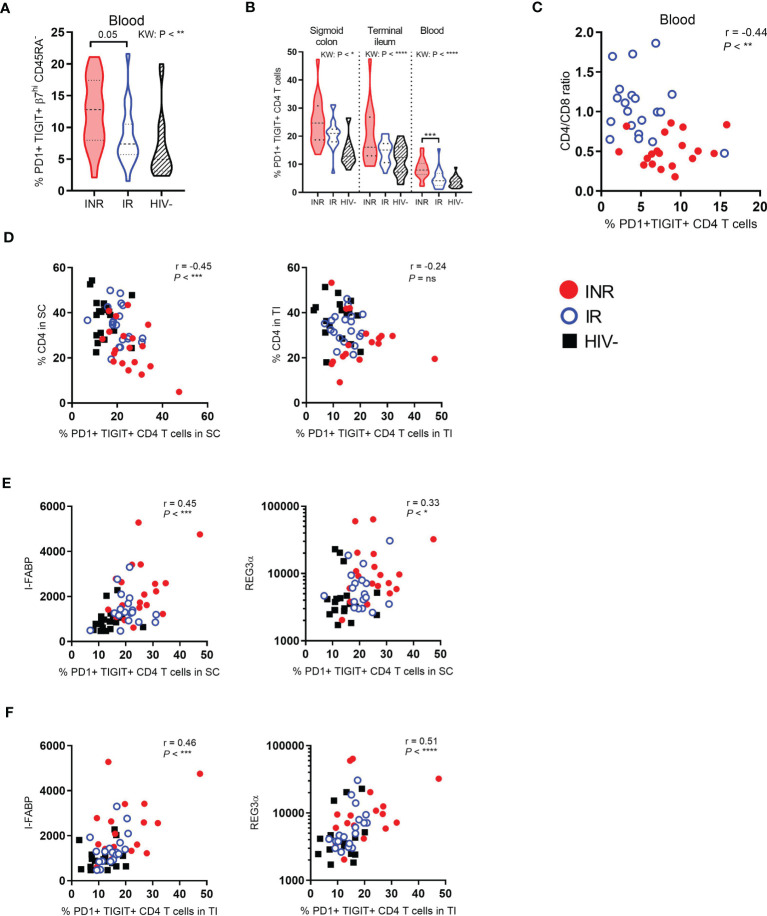
INR have higher fractions of exhausted CD4^+^ T cells in both blood and gut which correlates with markers of enterocyte damage. PD1 and TIGIT expression analyzed by flow cytometry on gut-homing CD4^+^ T cells in blood **(A)**, and on the total CD4^+^ T cell population in both gut and blood **(B)**. **(C)** Correlation between the CD4:CD8 ratio and PD1^+^ TIGIT^+^ CD4^+^ T cells in blood. **(D)** Correlation between the percentage of CD4^+^ T cells in sigmoid colon (left) or terminal ileum (right) and PD1^+^ TIGIT^+^ CD4^+^ T cells in sigmoid colon or terminal ileum, respectively. **(E, F)** Correlation between I-FABP (left) or REG3α (right) and the percentage of PD1^+^ TIGIT^+^ CD4^+^ T cells in sigmoid colon **(E)** or terminal ileum **(F)**. INR indicated as red violin plots/dots, IR as open blue violin plots/circles, HIV negatives as lined black violin plots/black squares (dashed lines: median, dotted lines: quartiles). Statistical analyses were performed by Kruskal Wallis (KW) test with Dunn’s post-test between INR and IR and Spearman correlation. *P < 0.05, **P < 0.01, ***P < 0.001, ****P < 0.0001, ns, not significant.

Next, PBMC and mucosal cells were analyzed by flow cytometry to study CD4^+^ T cell exhaustion defined by simultaneous cell surface expression of PD1 and TIGIT. Compared to IR, INR had a strong tendency of higher fractions of circulating gut-homing CD4^+^ T cells that co-expressed PD1 and TIGIT (*P* = 0.05, **Figure 2A**). Examining PD1 and TIGIT expression in the total CD4^+^ T cell populations in gut and blood, we found INR to have ubiquitously higher fractions of PD1^+^ TIGIT^+^ CD4^+^ T cells than IR and HIV negatives, although this was only statistically significant in the blood (**Figure 2B**). Additionally, in blood, the fraction of PD1^+^ TIGIT^+^ CD4^+^ T cells also correlated negatively with the CD4:CD8 ratio (r=-0.44, *P* < 0.01, **Figure 2C**). In gut mucosa, the fraction of PD1^+^ TIGIT^+^ CD4^+^ T cells in sigmoid colon correlated negatively with the fraction of CD4^+^ T cells in the same location (r=-0.45, *P* < 0.001, **Figure 2D**). Notably, no such correlation was detected in the terminal ileum.

We have previously shown that INR have higher levels of I-FABP and a tendency of higher levels of REG3α in plasma than IR (29). Importantly, the fractions of PD1^+^ TIGIT^+^ CD4^+^ T cells, in both sigmoid colon (r=0.45, *P* < 0.001 and r=0.3, *P* = 0.01, **Figure 2E**) and terminal ileum (r=0.46, *P* < 0.001 and r=0.51, *P* < 0.0001, **Figure 2F**), correlated with I-FABP and REG3α which suggests a pathogenetic relationship between epithelial damage and exhausted mucosal CD4^+^ T cells.

### CD4^+^ T Cell Subpopulations Are More Exhausted in INR, Both in Gut and Blood

**Figure 3:**
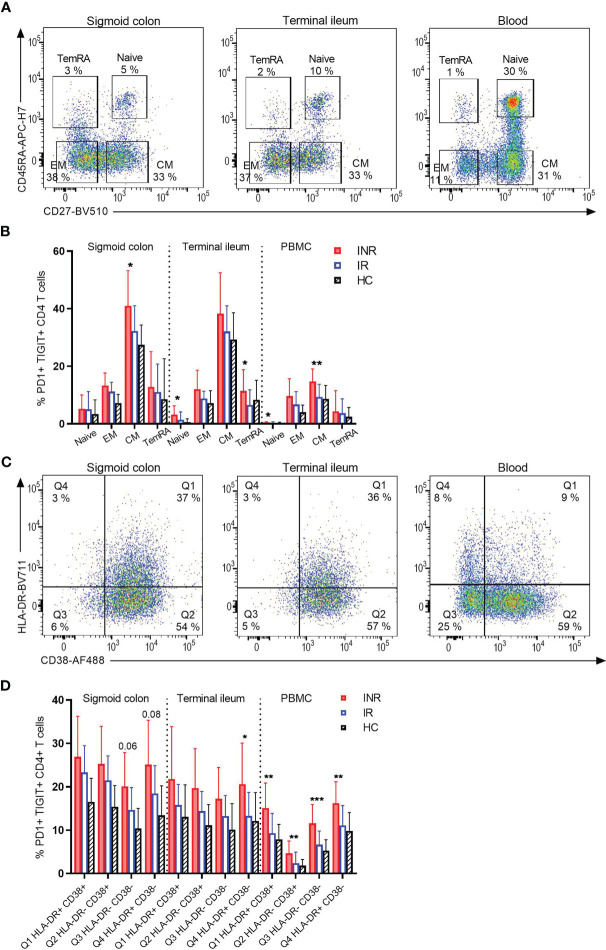
INR display more exhaustion of various CD4^+^ T cell subpopulations, both in gut and blood. **(A)** Representative flow cytometry plot of differentiated CD4^+^ T-cell subpopulations based on CD45RA and CD27 expression (naive: CD45RA^+^ CD27^+^, EM: CD45RA^-^ CD27^-^, CM: CD45RA^-^ CD27^+^, TemRA: CD45RA^+^ CD27^-^) in the three segments sigmoid colon, terminal ileum and blood of an INR. **(B)** Comparison of PD1^+^ TIGIT^+^ cells within each CD4^+^ T cell subpopulation in the three segments. **(C)** Representative flow cytometry plot of HLA-DR and CD38 expression in the three segments sigmoid colon, terminal ileum and blood of an INR. **(D)** Comparison of PD1^+^ TIGIT^+^ cells within each CD4^+^ T cell subpopulation of HLA-DR and CD38 expression in the three segments. INR (red), IR (open blue) and HIV negatives (black lined). Error bars indicate standard deviation. Statistical analyses were performed by Kruskal-Wallis (KW) test with Dunn’s post-test between INR and IR. *P < 0.05, **P < 0.01, ***P < 0.001.

To assess whether the observed higher PD1 and TIGIT expression on CD4^+^ T cells was restricted to a specific T cell differentiation stage, mucosal cells and PBMC were analyzed for CD45RA and CD27 expression to differentiate between naive (CD45RA^+^ CD27^+^), central memory (CM: CD45RA- CD27^+^), effector memory (EM: CD45RA- CD27-) and T effector memory cells re-expressing CD45RA (TemRA: CD45RA^+^ CD27-) (**Figure 3A**). Except for a tendency of INR to have lower fractions of naive CD4^+^ T cells in blood compared to IR, there were no differences in the other subpopulations in either of the three segments (data not shown). However, in both mucosal tissue and blood, INR had ubiquitously higher fractions of PD1^+^ TIGIT^+^ CD4^+^ T cell subpopulations compared to IR and HIV negatives (**Figure 3B**). Furthermore, INR displayed significantly more exhausted CD4^+^ T cells than IR for some subpopulations, such as CM in colon, naive and TemRA in ileum, and naive and CM in blood.

We then investigated by flow cytometry whether the high fractions of CD4^+^ T cells positive for PD1 and TIGIT in INR were restricted to activation status, as defined by HLA-DR^+^ CD38^+^ co-expression (**Figure 3C**). Overall, we found INR to have higher fractions of PD1^+^ TIGIT^+^ CD4^+^ T cells regardless of HLA-DR and CD38 expression. Hence, exhaustion of CD4^+^ T cells was ubiquitous and not restricted to CD4^+^ T cell activation (**Figure 3D**). This was apparent in both sigmoid colon and terminal ileum, as well as in blood. Yet, for several of the subpopulations in all three compartments INR showed higher fractions of PD1^+^ TIGIT^+^ cells when compared to IR specifically (**Figure 3D**).

### INR Have More Activated Treg in Terminal Ileum

**Figure 4:**
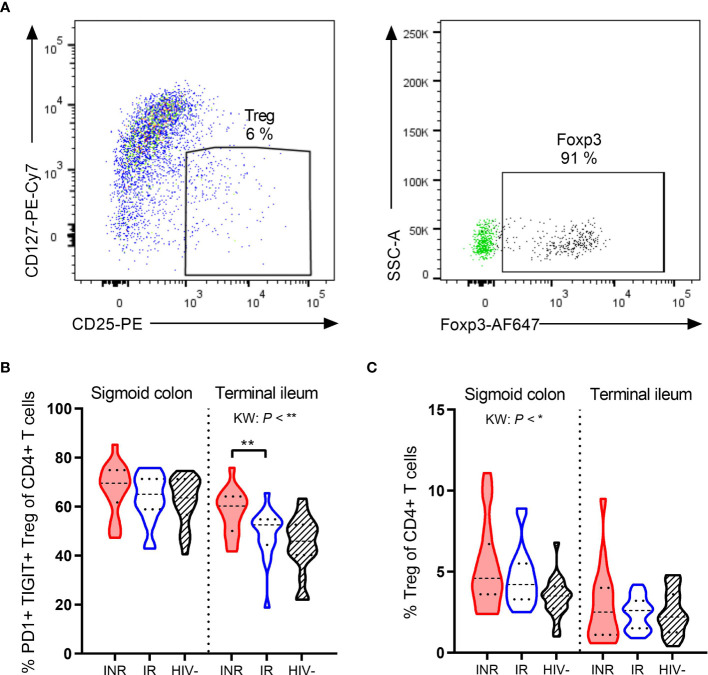
INR have higher fractions of PD1^+^ TIGIT^+^ Treg in terminal ileum. **(A)** Representative flow cytometry plot of regulatory T cells (Treg) from lamina propria mononuclear cells. In the right plot, fluorescence-minus-one control for Foxp3 (green dots) is overlaid the fully stained sample (black dots). **(B, C)** Comparison of the PD1^+^ TIGIT^+^ Treg **(B)** and percentages of Treg **(C)** and in sigmoid colon and terminal ileum in INR, IR and HIV negatives. INR indicated as red violin plots, IR as open blue violin plots, HIV negatives as lined black violin plots (dashed lines: median, dotted lines: quartiles). Statistical analyses were performed by Kruskal Wallis (KW) test with Dunn’s post-test between INR and IR. *P < 0.05, **P < 0.01.

The expression of PD1, TIGIT and other exhaustion markers on regulatory T cells (Treg) indicates Treg activation (30, 31). We defined Treg as CD127^low^ CD25^hi^ (90% of these cells were Foxp3^+^, **Figure 4A**) and assessed their expression of PD1 and TIGIT in gut lamina propria by flow cytometry. INR had higher fractions of PD1^+^ TIGIT^+^ Treg than IR (*P* < 0.01, **Figure 4B**) in the terminal ileum, but not in the sigmoid colon. However, this increased activation was not an indication of INR having higher fractions of Treg in gut mucosa as there was no difference in the percentages of total Treg between INR and IR (**Figure 4C**).

### Low Expression of PD-L1 in Gut Mucosa

**Figure 5:**
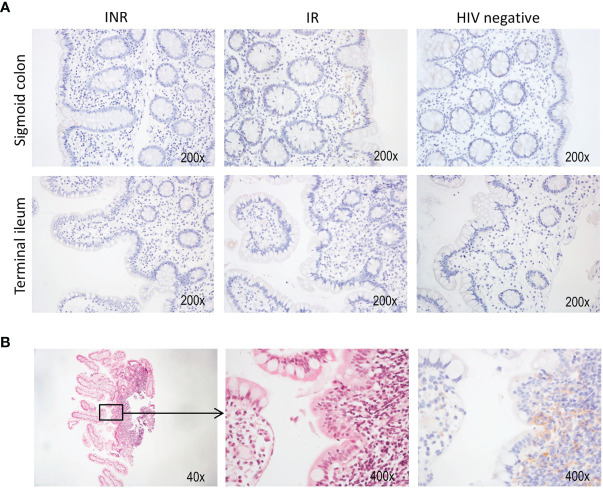
HIV infected patients lack PD-L1 expression in mucosa. Immunohistochemistry was performed on a selection of nine patients to assess the expression of PD-L1 in both sigmoid colon and terminal ileum. **(A)** Representative stainings from both anatomical sites in each patient group. **(B)** Hematoxylin-eosin (left and middle panel) and anti-PD-L1 (right panel) staining of a biopsy from the terminal ileum. This sample also contained tissue from a lymphoid follicle.

In the clinic, immunohistochemistry staining of PD-L1 is used to assess susceptibility to immune checkpoint inhibition by anti-PD1 or anti-PD-L1 in cancer patients (32). As we demonstrated that INR had more exhausted PD1^+^ TIGIT^+^ CD4^+^ T cells in the gut, we wanted to investigate whether PD-L1 was expressed in the gut mucosa. Mucosal pinch biopsies from three subjects of each study group were analyzed by immunohistochemistry to study the expression of PD-L1 in both sigmoid colon and terminal ileum. While PD-L1 was not overtly expressed within the regular lamina propria (**Figure 5A**), it was detected in a single sample from the terminal ileum of one INR subject that contained lymphoid follicles (**Figure 5B**).

## Discussion

In this study we found INR to have more gut-homing CD4^+^ T cells in blood than IR, and the fractions of gut-homing CD4^+^ T cells correlated negatively with CD4:CD8 ratio, indicating that the high level of gut-homing cells is an unfavorable prognostic marker in PLHIV. Second, INR had more exhausted gut-homing CD4^+^ T cells than IR, which was also seen for the total CD4^+^ T cell population in both blood and gut mucosa. The exhausted CD4^+^ T cells in INR were ubiquitous and could not be allocated to specific subsets of CD4^+^ T cell activation or differentiation but the degree of CD4^+^ T cell exhaustion correlated inversely with total CD4^+^ T cell fraction in colon Third, the fractions of exhausted mucosal CD4^+^ T cells correlated strongly with soluble markers of enterocyte damage. Our results add to the evidence linking gut mucosal damage and the observed immunopathogenesis in INR.

The increase of peripheral blood gut-homing CD4^+^ T cells in INR may be a consequence of increased priming and activation of mucosal T cells in this group, putatively due to an impaired mucosal barrier and increased microbial translocation of gut luminal antigens (3, 16). This notion is supported by recent reports demonstrating *ex vivo* that in HIV infection imprinting of α4β7 on CD4^+^ T cells is induced by retinoic acid (RA) produced by dendritic cells in response to toll-like receptor 2 stimulation of bacterial and fungal products (33, 34). An alternative explanation is that INR have an intensified homeostatic recruitment of CD4^+^ T cells to the gut in an attempt to repair the CD4^+^ T cell loss in this compartment. Studies have suggested that CD4^+^ T cell repopulation of the gut is maintained by homing in terms of cellular trafficking and not local cell proliferation (35, 36). We report a modest positive correlation between gut-homing CD4^+^ T cells in blood and the fractions of CD4^+^ T cells in sigmoid colon, which was apparent for both INR and IR, but not for HIV negative controls. A similar correlation was also found in the terminal ileum, but for INR only. Interestingly, we observed that the fractions of gut-homing CD4^+^ T cells correlated negatively with blood CD4:CD8 ratio. This indicates that even though increased gut-homing may be a part of an intrinsic attempt to resolve the CD4^+^ T cell loss in the gut, the increased recruitment was not associated with favorable T cell dynamics. Gp120 on HIV can bind to α4β7 on target cells to enable HIV entry into the cell (37, 38). α4β7^+^ T cells can also become activated upon binding Gp120, which can further increase the infection rate and replication rate of HIV (37, 39). Furthermore, in parallel to α4β7 induction, RA may induce a transcriptional program in CD4^+^ T cells increasing their permissiveness to HIV infection and viral replication (33, 34). Taken together, α4β7 may facilitate the mucosal transmission and maintenance of the HIV reservoir, which is shown to predispose for the INR phenotype (40, 41). A study by Sivro et al. reported that the frequency of α4β7^+^ memory cells pre-HIV infection was associated with HIV acquisition. The amount of α4β7^+^ memory CD4^+^ T cells pre-HIV infection was also found to be a predictor of the loss of CD4^+^ T cells below 500 cells/µl after HIV infection and a lower CD4:CD8 ratio (42). This supports an association between the high fractions of gut-homing CD4^+^ T cells and the low CD4:CD8 ratio we observed.

T cell exhaustion in PLHIV is associated with reduced HIV-specific T cell responses and increased HIV persistence and disease progression (24, 43). INR are reported to have more exhausted T cells (25, 26, 28). In line with this, we found the INR in our study to have higher fractions of exhausted CD4^+^ T cells. Interestingly, mucosal PD1^+^ TIGIT^+^ CD4^+^ T cells both in the sigmoid colon and the terminal ileum correlated positively with I-FABP and REG3α markers of enterocyte damage. Also, INR had higher fractions of exhausted gut-homing CD4^+^ T cells in the blood compared to IR. Together, these data support the hypothesis that mucosal damage and immune exhaustion are linked. Indeed, chronic stimulation of T cells due to reduced enterocyte function and microbial translocation, as well as residual HIV replication, may drive the T cells into an exhausted state that reduces the proliferative potential of the cells. This may be one of the reasons why INR are unable to increase their CD4^+^ T cell numbers.

In a recent publication we have reported that INR have a lower fraction of total mucosal CD4^+^ T cells in colon, but not in ileum (29). In line with this we here observed that exhausted CD4^+^ T cells correlated inversely with the fraction of total CD4^+^ T cells in colon while no correlation was detected in ileum. This raises the hypothesis that there is a colon-specific interaction between exhaustion status and density of CD4^+^ T cells, which deserves further investigations

We found higher levels of CD4^+^ T cell exhaustion in INR as an overall phenomenon regardless of activation or differentiation status. We have previously reported no difference in activation status of mucosal CD4^+^ T cells between INR and IR (29). In both gut compartments we now found the CM CD4^+^ T cells to exhibit the highest fraction of exhausted cells, with the percentages of this cell type higher in INR compared to IR. A study by Chomont et al. reported that memory cells with a low proliferative potential (PD1^+^ CM and transitional memory cells) contained more proviral DNA than EM and terminally differentiated cells, and suggested that these cells support latent HIV infection (44). We found INR to harbor more PD1^+^ TIGIT^+^ CM cells than IR in both the sigmoid colon and the blood. This raises the question whether INR have a more profound latent HIV infection and a larger viral reservoir than IR. Fromentin et al. showed that CD4^+^ T cells expressing the exhaustion markers PD1, TIGIT and LAG3, separately or in combination, also are enriched for inducible HIV genomes and that their presence is strongly associated with the size of the HIV reservoir (43). This finding was even stronger in exhaustion marker-expressing memory cells. Regarding our definition of exhausted T cells, functional assays were not conducted to confirm the exhaustion of the CD4^+^ T cells. However, it is shown that exhausted T cells usually express several immune checkpoint markers (45, 46). To ensure specificity, we defined exhausted cells as CD4^+^ T cells double positive for PD1 and TIGIT.

Treg may reduce the chronic inflammation observed in PLHIV. On the other hand, Treg may also dampen HIV-specific immune responses, as well as the proliferation of CD4^+^ T cells (47). By comparing mucosal Treg in all three study groups, we found the two PLHIV groups to have higher fractions of Treg in the sigmoid colon, but not in the terminal ileum, compared with HIV negative controls. However, studying PD1 and TIGIT expression on Treg, we found INR to have higher fractions of this cell population in the terminal ileum than IR. Treg expressing PD1 and TIGIT have been shown to be highly suppressive (activated), and not exhausted (30, 31, 48). Hence, the higher fractions of activated Treg in the terminal ileum in INR is in concordance with our previous observations that there are more prominent immune alterations correlated to enterocyte damage in the sigmoid colon than in the terminal ileum of INR (29).

Immune checkpoint inhibitors are currently being used to treat cancer in PLHIV, and positive side effects such as reducing the HIV reservoir have been observed (49, 50). The use of immune checkpoint inhibitors in the HIV setting is appealing as it may reverse HIV latency, increase HIV-specific T cell responses and reverse the unresponsiveness of exhausted T cells. We could not detect any PD-L1 on cells within the regular lamina propria tissue, either in INR, IR or HIV negative controls in our immunohistochemistry analysis of gut mucosal tissues in a limited number of patients. On one side, a lack of PD-L1 may facilitate priming, activation and exhaustion in INR. On the other side, PD-L1 expression may be more important in GALT and could be related to decreased proliferation of CD4^+^ T cells in INR. However, the heterogeneity of the mucosal biopsies examined, in addition to the small sample size examined, warrants a more focused approach to address this issue.

Tissue heterogeneity is a potential caveat to the interpretation of flow cytometry data. The dissolved cells consisted primarily of lamina propria cells but would also include a minor fraction of intraepithelial lymphocytes. Some biopsies may consist purely of lamina propria tissue whereas other biopsies may contain GALT with a respective lower or higher immune cell content. In our study, we combined 20 biopsies from each segment before performing flow cytometry analysis. Pooling multiple biopsies should have reduced the sample heterogeneity.

Our study has some limitations. The statistical power of the study was limited, and some of the variables did not achieve statistical significance, even though there were visually apparent differences between median values in the INR and IR. We identified the gut-homing T cells by the high expression of ITGβ7 and lack of expression of CD45RA. In line with other reports (42), we confirmed that the cells that stained positive for ITGβ7 almost exclusively co-expressed ITGα4 (data not shown). CCR9, which indicates small intestine gut-homing, could have been included to fine-tune the results. In addition, excluding all cells that were positive for CD45RA could have resulted in us missing out on the analysis of gut-homing TemRA cells. Also, smaller numbers of γδ T cells, mucosal associated invariant T cells and natural killer T cells may be present in the mucosal CD4^+^ T cell population analyzed, as such unconventional T cells were not excluded by the gating strategy (**Figure S2**). Finally, all flow cytometry data are presented as fractions. Pilot studies that we performed showed us that weighing biopsies to normalize data to the weight of the biopsies was not feasible.

There are several strengths of our study. Such well-defined human study groups and the extensive mucosal sampling from *two* anatomical sites in addition to blood are rare in human INR studies. The CD4 threshold that defines INR varies in HIV research. In our study we used <400 cells/µl to define the INR group, which lays in the upper area for INR characterization. More importantly, included subjects had been on ART for more than four years, as the CD4 count rises most in the first years after ART initiation (51). The INR group had a substantially lower CD4:CD8 ratio than the IR group, adding validity to our INR group as factual INR with a poorer clinical prognosis than IR. Importantly, the INR and IR groups were matched on age and nadir CD4, two factors known to be confounders of the INR phenotype. Hence, the differences found in our study were not biased by these known confounders.

To conclude, this study supports the hypothesis that INR have a more exhausted CD4^+^ T cell pool than IR. The correlation between mucosal T cell exhaustion and enterocyte damage, and the link with increased gut-homing provide new insight into the INR phenotype in particular and for PLHIV in general. Further studies are warranted to explore why INR have more gut-homing CD4^+^ T cells without restoring the CD4^+^ T cell numbers in the gut, and whether this promotes the chronic disease by recruiting more cells to ameliorate the systemic inflammation while serving as targets for the virus.

## Data Availability Statement

The raw data supporting the conclusions of this article will be made available by the authors, without undue reservation.

## Ethics Statement

The studies involving human participants were reviewed and approved by Regional Committee for Medical and Health Research Ethics (approval id 2015/2125). The patients/participants provided their written informed consent to participate in this study.

## Author Contributions

KL, KT, and DR designed the study in collaboration with BS, AD-R, and DK. MM-M and AM included and examined all study participants. KL, MM-M, KK, CH, and ML-I performed laboratory analyses. KL, MM-M, KT, and DR analyzed the data. KL, MM-M, KT, and DR drafted the manuscript. All authors contributed to the article and approved the submitted version.

## Funding

This work was supported by grants from the South-Eastern Norway Regional Health Authority (grant no. 2013074 to KT and grant no. 2016018 to DK), the Research Council of Norway (grant no. 187615 to KT), Såstads Legat (to KL and MM-M), Stiftelsen Kristian Gerhard Jebsen (K.G. Jebsen Inflammation Research Centre) (to DR), Gilead Sciences Nordic Fellowship Programme 2017 (to DR) and Pasteurlegatet (to MM-M).

## Conflict of Interest

The authors declare that the research was conducted in the absence of any commercial or financial relationships that could be construed as a potential conflict of interest.

## Publisher’s Note

All claims expressed in this article are solely those of the authors and do not necessarily represent those of their affiliated organizations, or those of the publisher, the editors and the reviewers. Any product that may be evaluated in this article, or claim that may be made by its manufacturer, is not guaranteed or endorsed by the publisher.
