# Supplementary material for: Enhanced Gut-Homing Dynamics and Pronounced Exhaustion of Mucosal and Blood CD4+ T Cells in HIV-Infected Immunological Non-Responders
Source: Front Immunol. 2021 Oct 7;12:744155. doi: 10.3389/fimmu.2021.744155 (PMC8529151; doi:10.3389/fimmu.2021.744155)
Supplement: Supplementary file 1 [file DataSheet_1.pdf]

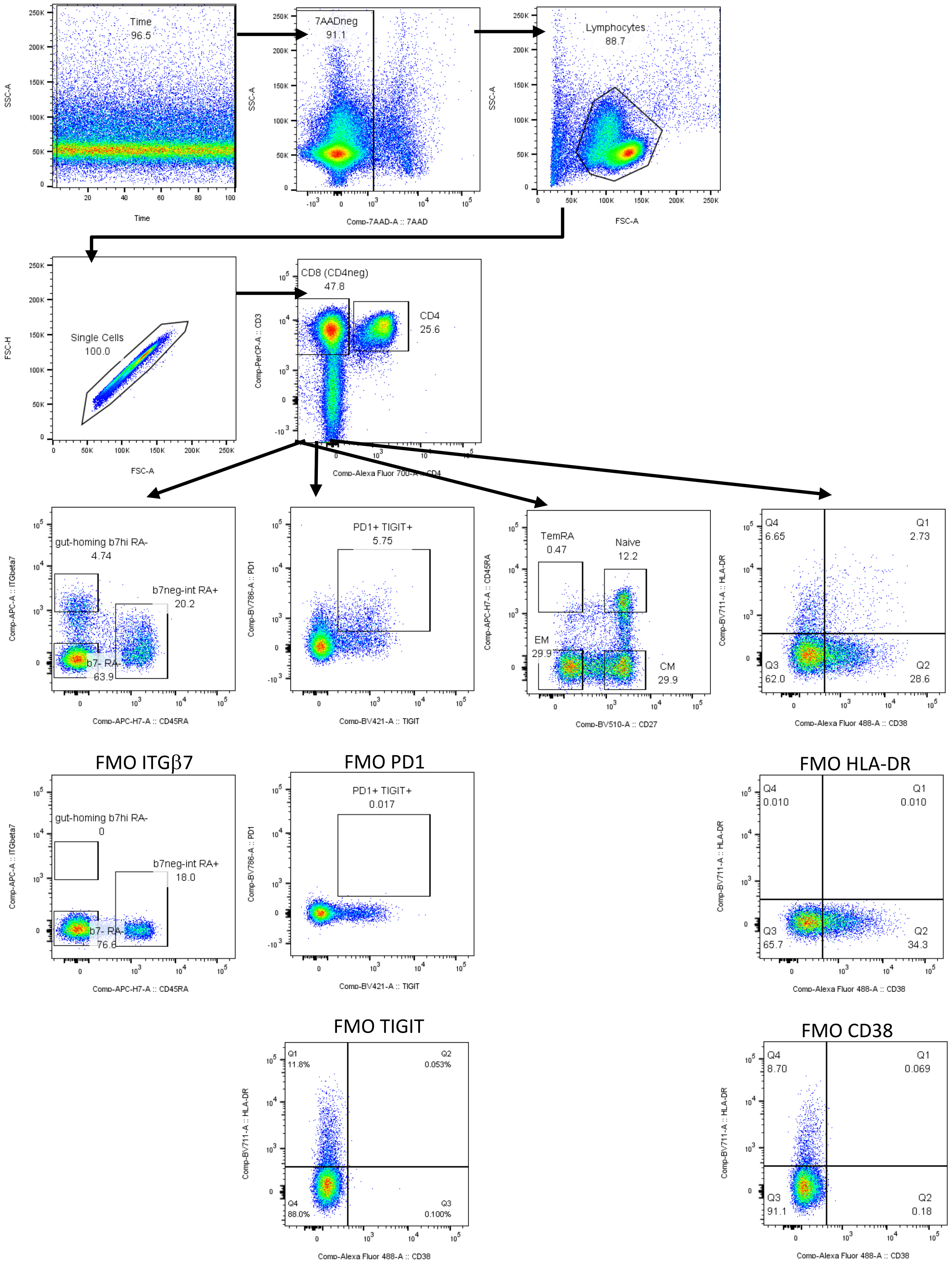

Figure S1 Flow cytometry gating strategy for PBMC. Fluorescence minus one (FMO) controls were used to set the gates of positive events for some of the surface markers.

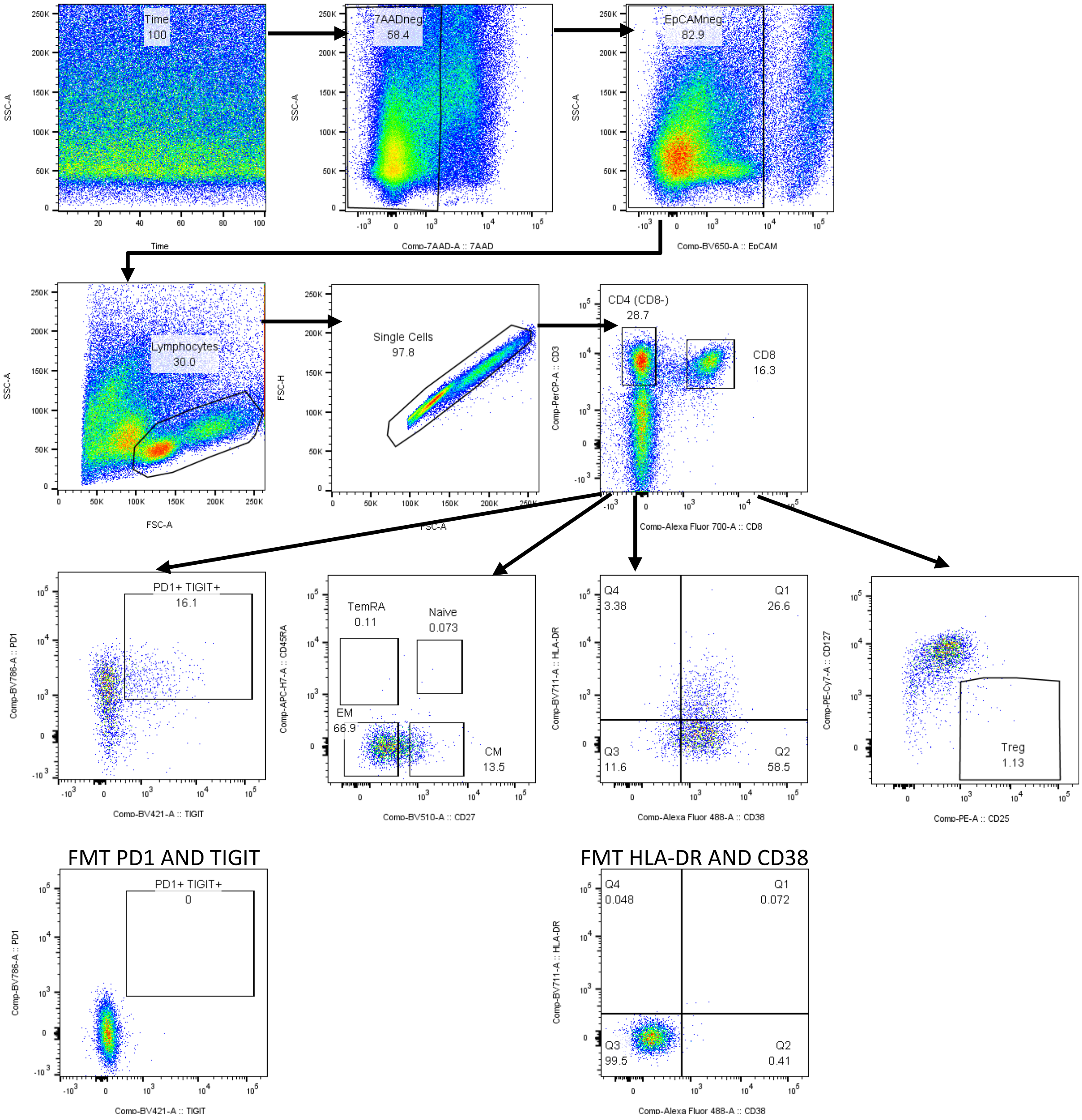

Figure S2 Flow cytometry gating strategy for mucosal cells. Fluorescence minus two (FMT) controls were used to set the gates of positive events for some of the surface markers.

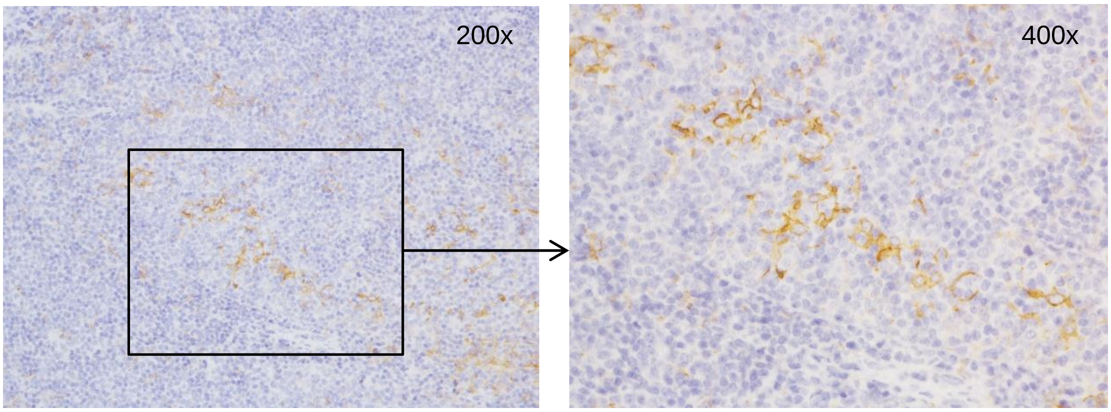

Figure S3 Positive staining of PD-L1 in tonsil control tissue.
